# Identification and Removal of a Fractured Portacath

**DOI:** 10.1155/crra/6411769

**Published:** 2026-04-16

**Authors:** Azri Johari, David Ding, Deepak Jain

**Affiliations:** ^1^ Faculty of Medicine, The University of Queensland, Herston, Queensland, Australia, uq.edu.au; ^2^ Medical Imaging Department, Redcliffe Hospital, Redcliffe, Queensland, Australia, health.qld.gov.au; ^3^ Medical Imaging Department, Westmead Hospital, Westmead, New South Wales, Australia, nsw.gov.au; ^4^ Medical Imaging Department, The Prince Charles Hospital, Chermside, Queensland, Australia, qld.gov.au

**Keywords:** case report, foreign body removal, fractured portacath, interventional radiology, percutaneous retrieval, venous access device

## Abstract

A 31‐year‐old woman with metastatic breast cancer was referred for chest radiography after portacath malfunction. Imaging identified an incomplete catheter fracture at the internal jugular vein that subsequently progressed to complete fracture during removal. Lodged in the superior vena cava, the fractured fragment was retrieved using a loop snare via femoral vein access under fluoroscopic guidance. Postretrieval imaging confirmed no residual fragments, and the patient had an uneventful recovery. Though rare, portacath fractures have associated life‐threatening complications such as vessel perforation, embolisation, cardiac tamponade and death. This case underscores the importance of early identification of portacath fractures and demonstrates interventional radiology techniques as a safe and precise method for their removal.

## 1. Introduction

Implantable venous access devices (IVAD), also known as port‐a‐catheters or simply as portacaths, are widely utilised to provide long‐term vascular access for patients requiring chemotherapy, parenteral nutrition or frequent blood sampling. Their design allows for repeated access while minimising the risks of infection and vascular damage compared with peripheral lines or peripherally inserted central catheters [1–3]. Recent international guidelines, including those from the Japanese Society of Interventional Radiology, emphasise that although portacaths are generally safe, mechanical complications such as catheter fracture remain clinically significant events that require prompt recognition and intervention [1].

Catheter fractures can result in migration of fragments, posing significant risks such as vessel perforation, embolisation, cardiac tamponade and sudden death [3, 4]. The incidence of mechanical portacath complications is around 4.3%, with fracture and migration reported to occur in only 0.1%–1% of cases [5–7]. Management includes percutaneous transcatheter removal, surgical open thoracotomy or long‐term warfarin therapy [8].

We report a case of a partially fractured polyurethane portacath that subsequently fractured completely perioperatively. This case highlights the importance of timely diagnosis and prompt intervention in mitigating potentially life‐threatening complications, demonstrating interventional radiological approaches as minimally invasive, safe and effective.

## 2. Case Presentation

**Figure 1:**
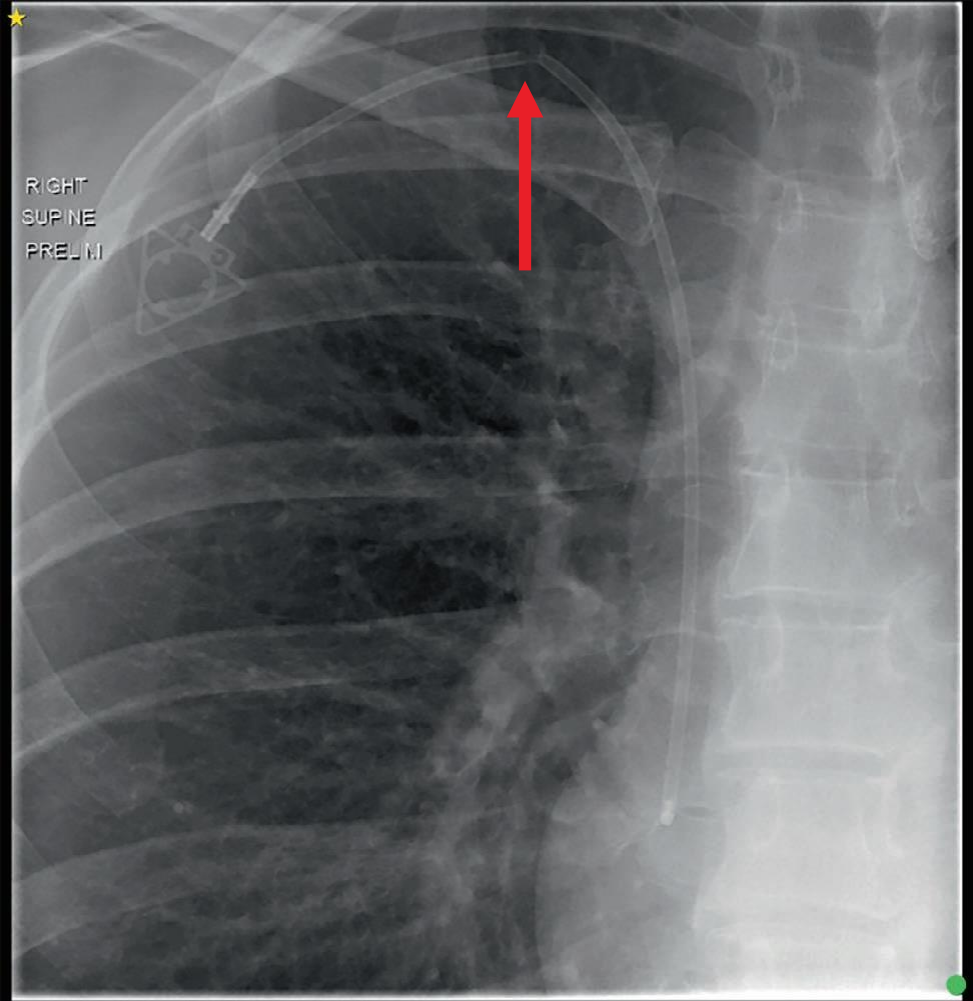
Chest radiograph demonstrating an incomplete fracture of the portacath at the internal jugular vein entry site (arrow).

A 31‐year‐old woman with a history of metastatic breast cancer was referred for imaging to evaluate the structural integrity of her right‐sided portacath following failure to flush or aspirate the device. An 8‐Fr Attachable ChronoFlex Polyurethane PowerPort had been inserted 3 years earlier via the right internal jugular vein for chemotherapy administration. Chest radiography revealed an incomplete fracture of the catheter at the entry point of the internal jugular vein (Figure 1). Her most recent imaging was a CT chest 2 years prior, which at the time, did not demonstrate any warning signs of impending fracture. Given the risk of complete fracture and associated complications, interventional radiology was consulted for removal of the portacath.

**Figure 2:**
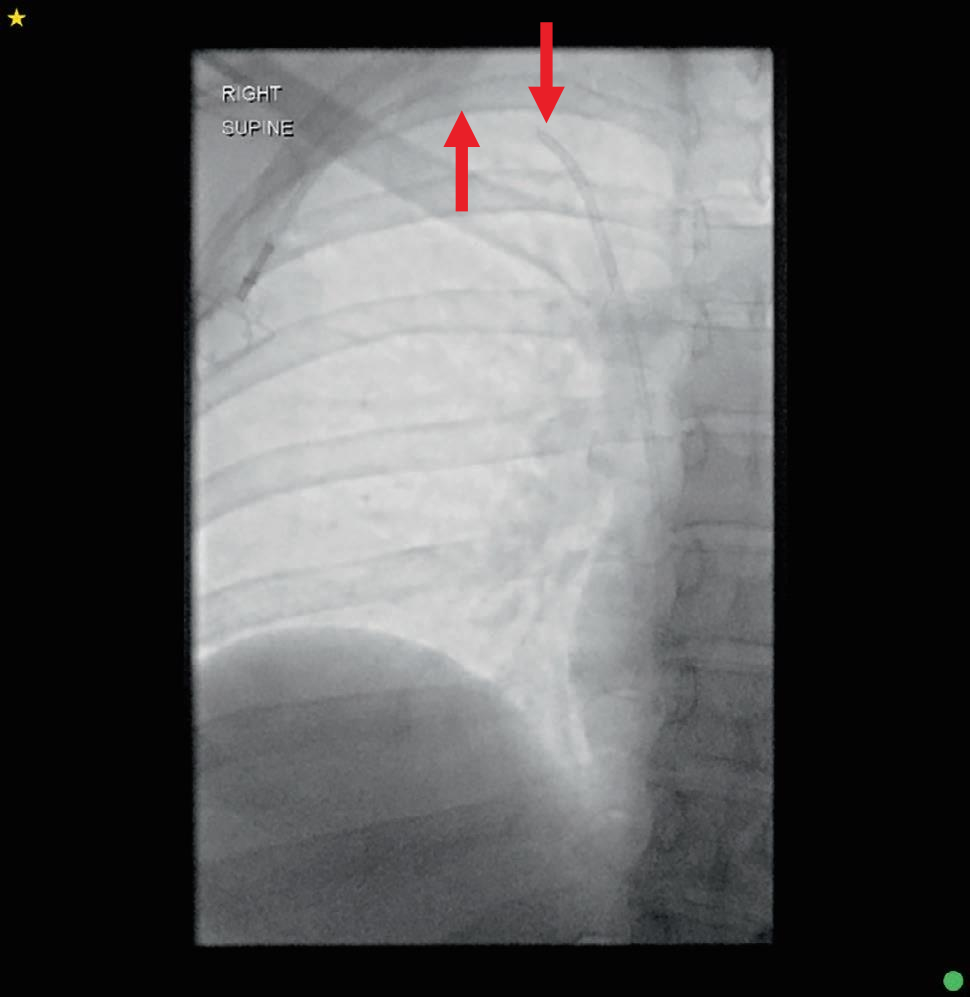
Perioperative fluoroscopic imaging now revealing complete fracture of the catheter (arrows).

Surgical removal of the port chamber and catheter was initially attempted, given the partially fractured catheter remained tethered to the subcutaneous port system. This approach would allow for controlled removal of the accessible component while minimising the risk of embolisation of the distal fragment. Under fluoroscopic guidance, the position of the portacath was established, confirming an angled partial fracture near the entry site of the internal jugular vein. Under conscious sedation and local anaesthetic, following standard sterile precautions, a neck incision near the fracture site and an incision at the port site were made. Despite careful subcutaneous dissection, the catheter could not be palpated at the neck, and further fluoroscopic imaging revealed a complete fracture of the catheter (Figure 2). The port chamber and the proximal fragment of the fractured catheter were dissected and removed from the port site, followed by subcutaneous and subcuticular suturing at the incision sites.

The distal fragment of the catheter remained lodged in the superior vena cava, and femoral vein access was utilised for its retrieval. Under ultrasound guidance, the right femoral vein was accessed using Seldinger technique; a 0.035‐in. hydrophilic guidewire and diagnostic catheter were advanced, followed by progressive dilatation up to a 9‐Fr sheath (Cook Medical). A 30‐mm Amplatz Goose Neck loop snare (Medtronic) was advanced via the inserted sheath into the right atrium and superior vena cava, successfully grasping the distal catheter fragment. The fractured fragment was then cautiously withdrawn and removed through the sheath. (Figures 3a, 3b, 3c, 3d, 3e and 3f).

Figure 3A sequence of fluoroscopic images showing a step‐by‐step retrieval of the fractured portacath: (a) femoral vein access via dilator sheath and 30‐mm Amplatz Goose Neck loop snare (Medtronic) insertion, (b) careful alignment of the snare with the catheter fragment, (c) successful grasping of the fragment and (d–f) withdrawal and removal of the fragment through the inserted sheath.

**Figure:**
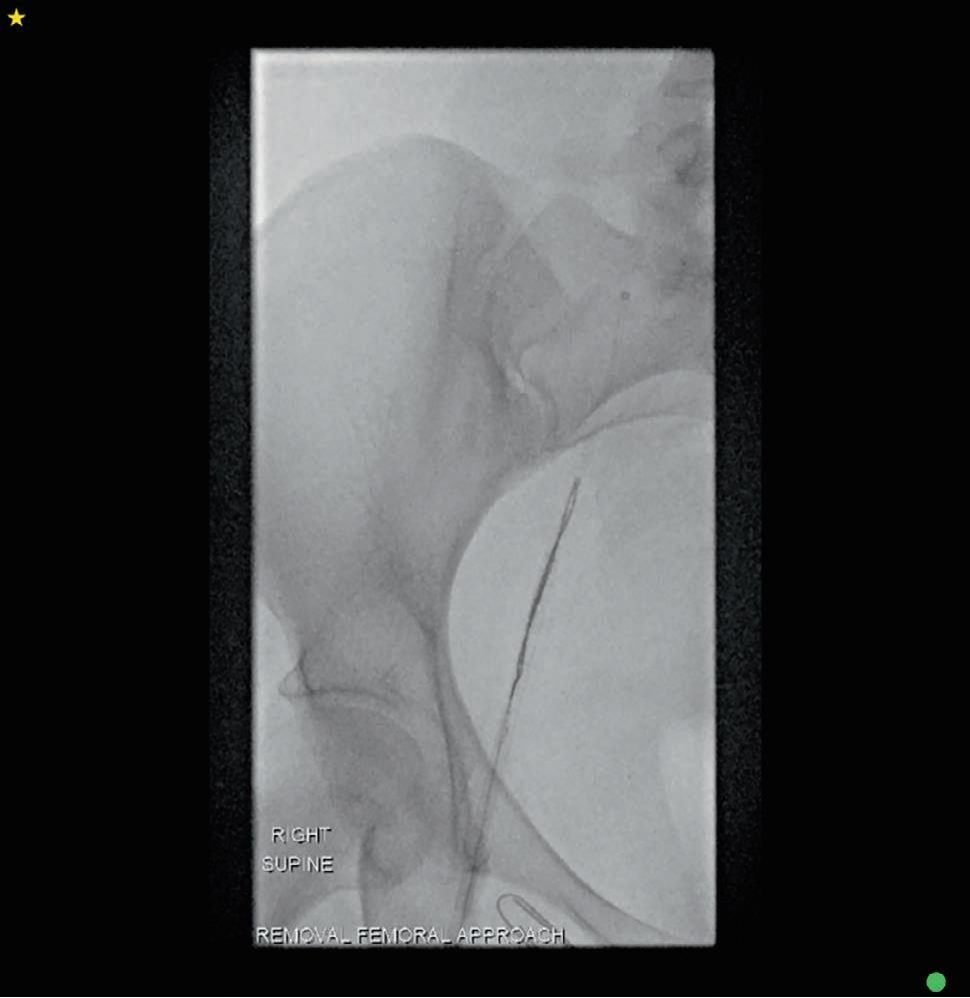
(a)

**Figure:**
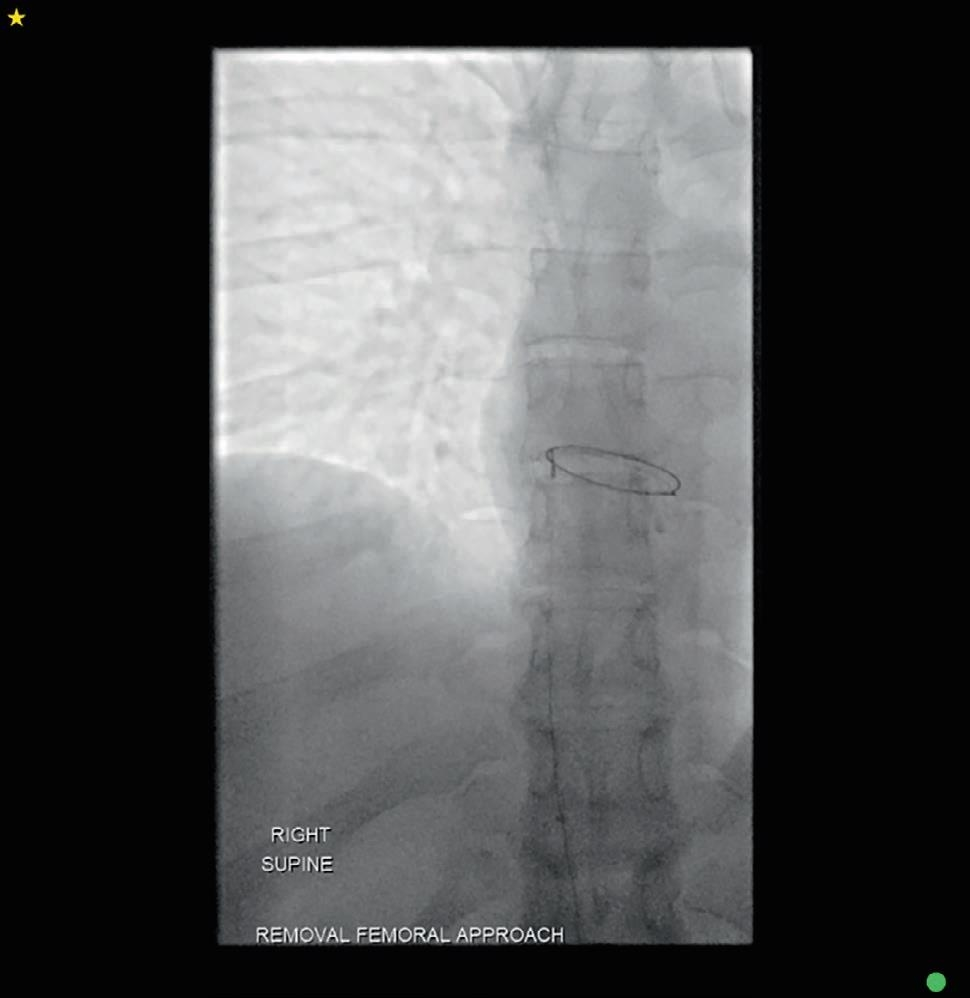
(b)

**Figure:**
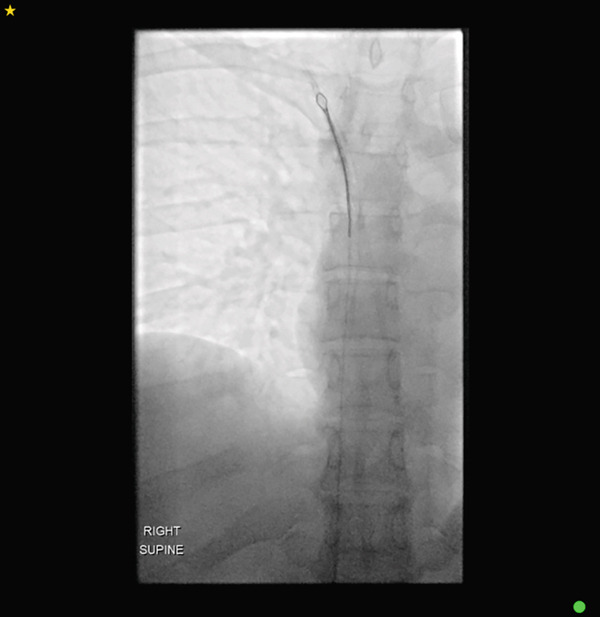
(c)

**Figure:**
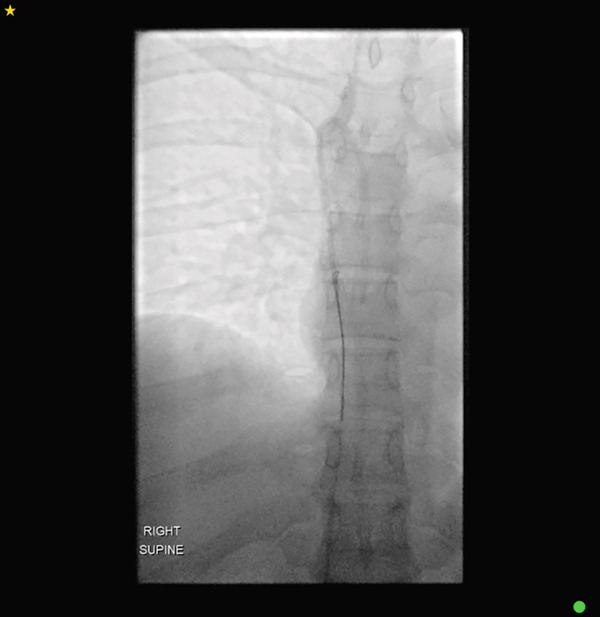
(d)

**Figure:**
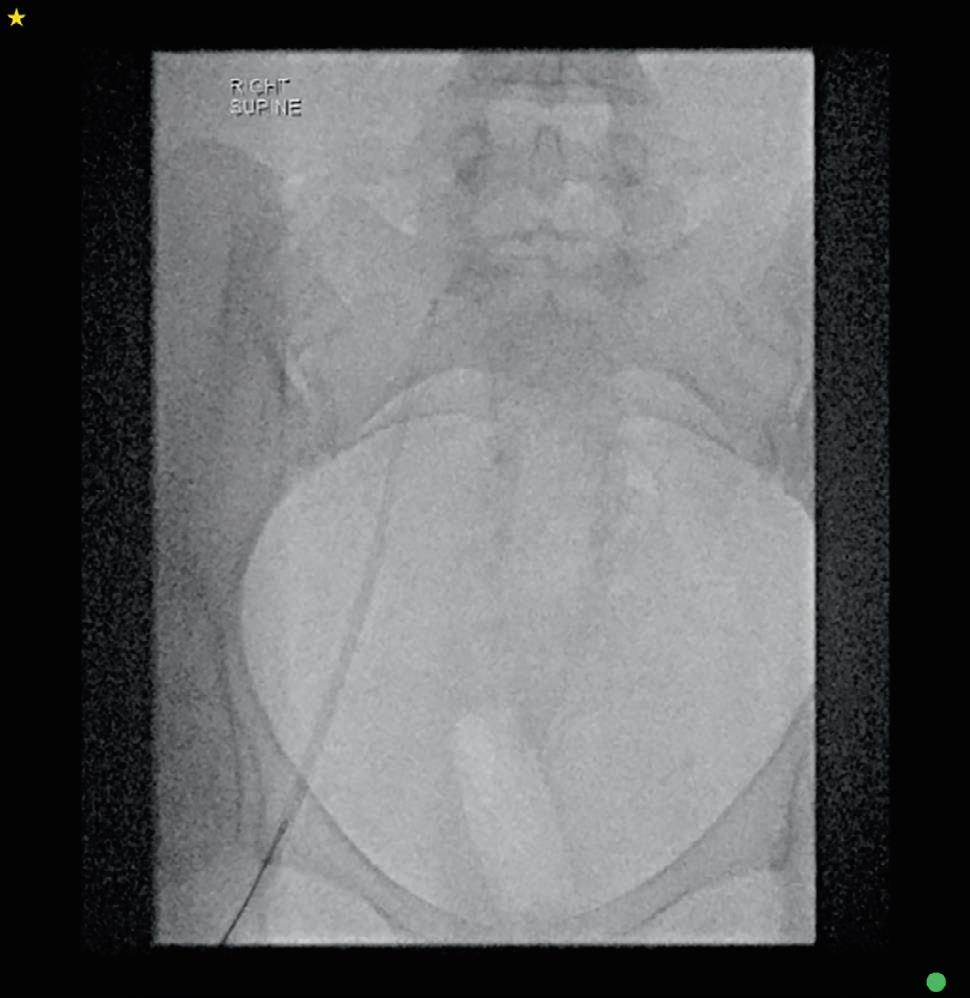
(e)

**Figure:**
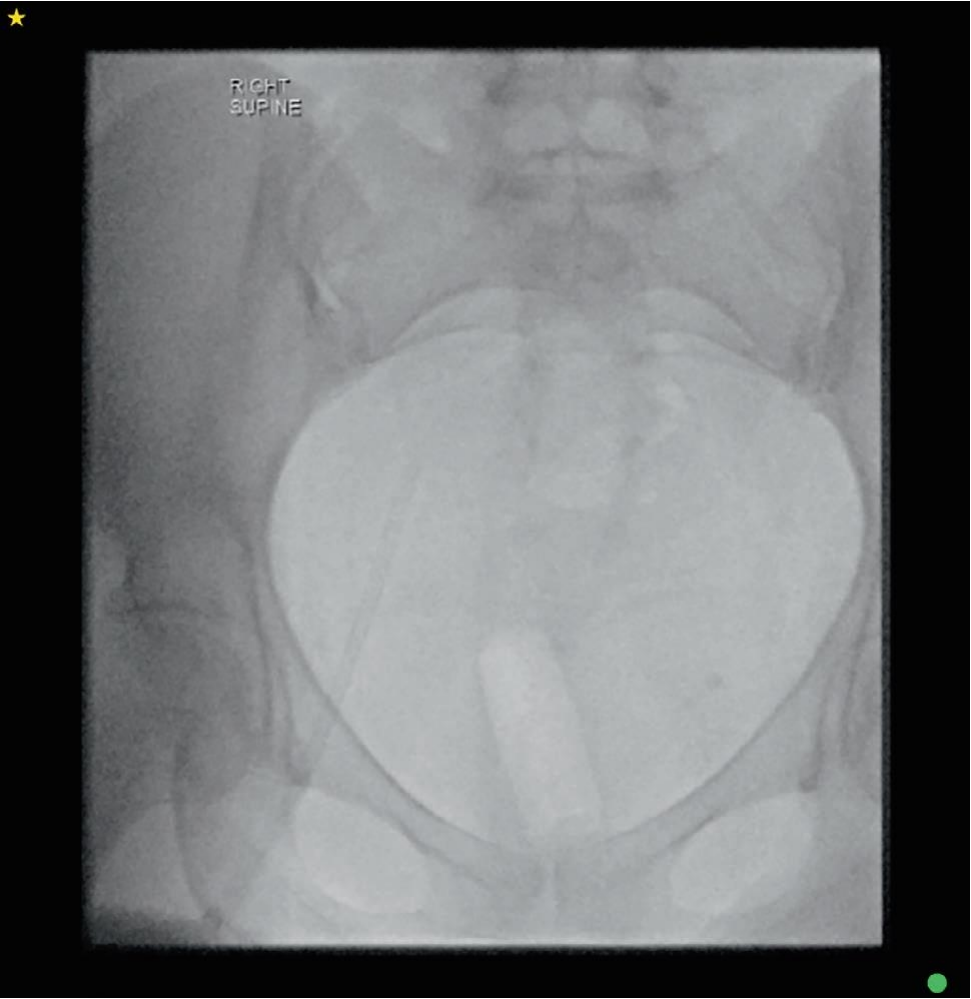
(f)

**Figure 4:**
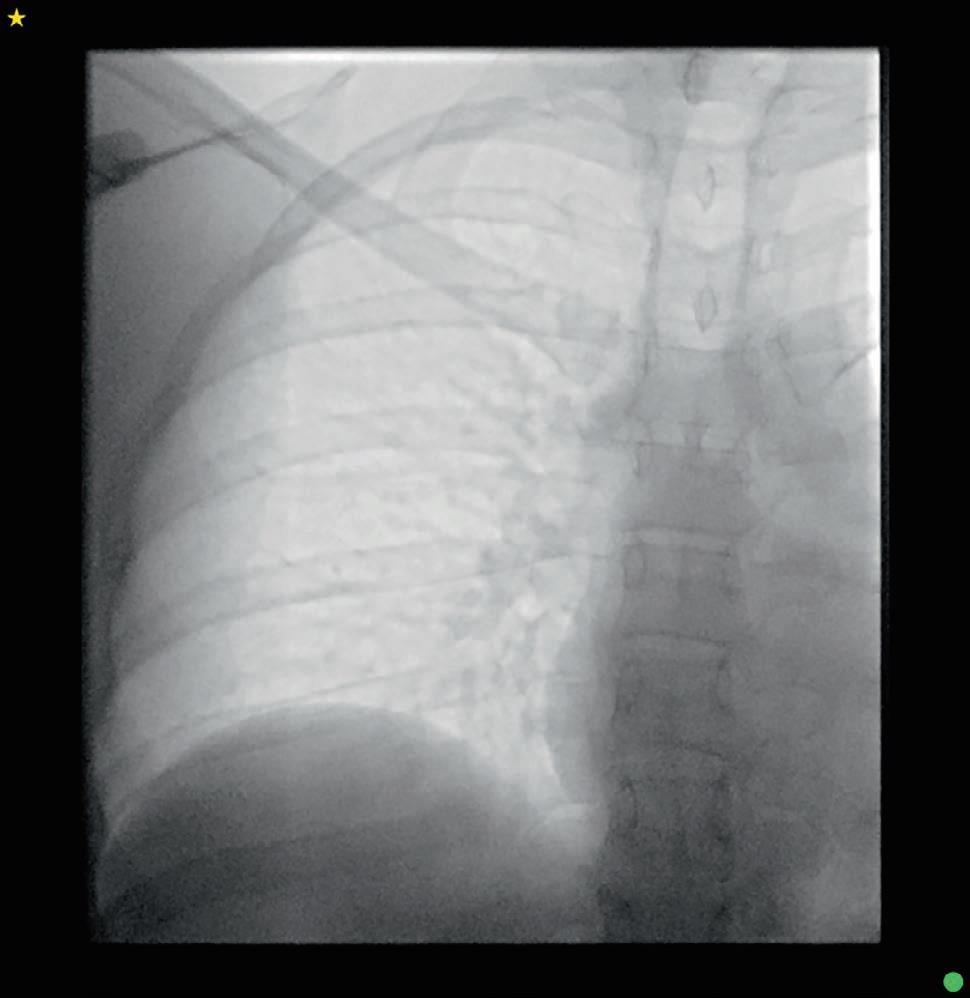
Fluoroscopic assessment ensuring no residual catheter fragments.

A final fluoroscopic assessment confirmed the absence of any residual catheter fragments within the body (Figure 4). The femoral sheath was subsequently removed, and haemostasis was achieved with manual compression and the puncture site was dressed. The patient tolerated the procedure and was monitored postprocedure with serial observations, and the femoral access site was assessed for bleeding and haematoma formation. After 2 hours of observation with stable clinical parameters, the patient was discharged home on the same day. No prophylactic anticoagulation was administered. The patient was reviewed in the outpatient clinic in the following weeks with no postprocedural complications.

## 3. Discussion

Portacath complications may arise during the insertion procedure or during the maintenance period. Infection‐related complications requiring removal are more frequently reported and can potentially lead to serious outcomes such as bacteraemia or septic emboli [1–6, 9, 10]. Two large retrospective cohort studies reported between 2.3% and 3.2% of portacath insertions had portacath‐related infections [5, 6]. Noninfectious complications requiring removal occur at a lower incidence, and include venous thromboembolism, catheter impingement, catheter occlusion and catheter fracture and migration [1–4, 6]. Factors which may contribute to portacath fracture and migration include excessive intracatheter pressure via repetitive flushing using a small‐volume syringe, external compression such as from seat belts or jewellery, and material failure exacerbated by sharp angle formation during the implantation of the portacath [1, 5, 9].

The material composition of portacaths also influences mechanical durability. Polyurethane catheters possess greater mechanical resistance to fatigue and compression, whereas silicone catheters are more flexible but have lower tensile strength. Several studies have demonstrated higher rates of silicone portacath fracture when compared with polyurethane devices [11–13]. However, as illustrated in this case, polyurethane portacaths remain susceptible to fracture and associated complications.

Symptoms of catheter fracture and migration may present with swelling, infraclavicular pain, shoulder pain, chest pain, or palpitations. However, symptoms are often nonspecific or absent, with port malfunction often being the first sign of portacath fracture, prompting further investigation [5, 6]. If there is complete fracture of the catheter, the fragmented catheter may migrate to the right atrium and ventricle, pulmonary arteries or the superior/inferior vena cava [10]. Given fractured portacaths can give rise to life‐threatening complications, prompt removal of both partial and complete fractured portacaths is recommended to ensure patient safety [1, 4, 5].

Compared with surgical removal of intravascular foreign bodies, interventional radiological approaches offer significant advantages such as lower morbidity, shorter hospital stays, and reduced anaesthesia‐related risks [14]. Advances in interventional radiology have introduced an extensive range of specialised devices, including snares, baskets and graspers, tailored for extracting foreign bodies from both vascular and nonvascular sites [8, 9]. Under fluoroscopic guidance, Cheng et al. reported a 97.8% success rate with a low complication rate of 3.3% for fractured catheter retrievals via femoral vein approach using an 8‐Fr vascular sheath. Their findings highlighted the reliability of a variety of devices, with loop snares being used in 67.0% of cases [8]. Loop snares are often regarded as the first‐line device for intravascular foreign body removal, and our case further demonstrates their reliability and precision [10, 14, 15].

## 4. Strengths and Limitations

As a single case report, the findings may not be generalisable; however, this case highlights an uncommon but clinically significant complication of long‐term polyurethane portacath use and demonstrates the effectiveness of endovascular retrieval.

## 5. Conclusion

Portacath fractures are often asymptomatic and may simply present as difficulty flushing or aspirating of the patient′s portacath. Despite their subtle presentation, portacath fractures pose significant life‐threatening risks, where timely identification and removal of fractured fragments is crucial. Interventional radiology approaches are minimally invasive and have been shown to safely and effectively mitigate these associated complications.

## Funding

No funding was received for this manuscript. Open access publishing facilitated by The University of Queensland, as part of the Wiley ‐ The University of Queensland agreement via the Council of Australasian University Librarians.

## Consent

Consent was obtained from the patient for the publication of this case report in a deidentified manner for academic purposes.

## Conflicts of Interest

The authors declare no conflicts of interest.

## Supporting information


**Supporting Information** Additional supporting information can be found online in the Supporting Information section. File S1: CARE checklist.

## Data Availability

Data sharing is not applicable to this article as no datasets were generated or analysed during the current study.
